# Stimulation of Autotrophic Denitrification by Intrusions of the Bosporus Plume into the Anoxic Black Sea

**DOI:** 10.3389/fmicb.2012.00257

**Published:** 2012-07-18

**Authors:** Clara A. Fuchsman, James W. Murray, James T. Staley

**Affiliations:** ^1^School of Oceanography, University of WashingtonSeattle, WA, USA; ^2^Department of Microbiology, University of WashingtonSeattle, WA, USA

**Keywords:** Black Sea, autotrophic denitrification, *Sulfurimonas*, Bosporus Plume, anammox

## Abstract

Autotrophic denitrification was measured in the southwestern coastal Black Sea, where the Bosporus Plume injects oxidized chemical species (especially O_2_ and ${\text{NO}}_{\text{3}}^{-}$) into the oxic, suboxic, and anoxic layers. Prominent oxygen intrusions caused an overlap of ${\text{NO}}_{\text{x}}^{-}$ and sulfide at the same station where autotrophic denitrification activity was detected with incubation experiments. Several bacteria that have been proposed to oxidize sulfide in other low oxygen environments were found in the Black Sea including SUP05, *Sulfurimonas*, *Arcobacter*, and BS-GSO2. Comparison of TRFLP profiles from this mixing zone station and the Western Gyre (a station not affected by the Bosporus Plume) indicate the greatest relative abundance of *Sulfurimonas* and *Arcobacter* at the appropriate depths at the mixing zone station. The autotrophic gammaproteobacterium BS-GSO2 correlated with ammonium fluxes rather than with sulfide fluxes and the maximum in SUP05 peak height was shallower than the depths where autotrophic denitrification was detected. Notably, anammox activity was not detected at the mixing zone station, though low levels of DNA from the anammox bacteria *Candidatus* *Scalindua* were present. These results provide evidence for a modified ecosystem with different N_2_ production pathways in the southwest coastal region compared to that found in the rest of the Black Sea. Moreover, the same *Sulfurimonas* phylotype (BS139) was previously detected on >30 μm particles in the suboxic zone of the Western Gyre along with DNA of potential sulfate reducers, so it is possible that particle-attached autotrophic denitrification may be an overlooked N_2_ production pathway in the central Black Sea as well.

## Introduction

Three processes are responsible for N_2_ production under low oxygen conditions: (1) heterotrophic denitrification, which converts nitrate to N_2_ using organic matter as a reductant; (2) anammox, an autotrophic process which reduces nitrite with ammonium to form N_2_; and (3) autotrophic denitrification, which converts nitrate to N_2_ using reduced sulfur species as a reductant. In both heterotrophic and autotrophic denitrification, nitrate is reduced using the same pathway with N_2_O as an intermediate product (Sievert et al., 2008). Autotrophic denitrification has been found to be an important N_2_ production pathway in anoxic water columns in the Baltic Sea (Hannig et al., 2007), the Benguela upwelling zone (Lavik et al., 2009), and Mariager Fjord, Denmark (Jensen et al., 2009).

The Black Sea is a permanently anoxic basin with a well-defined redox gradient. A 20- to 80-km wide rim current circulates around the perimeter of the Black Sea, enclosing two cyclonic gyres (Poulain et al., 2005). In most of the Black Sea, the Cold Intermediate Layer, with a characteristic core density of σ_θ_ ≈ 14.5, represents the lower boundary of direct communication with the surface. The suboxic zone lies between the oxic Cold Intermediate Layer and a 2000-m thick sulfidic zone. In the central Black Sea, autotrophic denitrification is generally not thought to be important. Anammox has been detected in the suboxic zone (Kuypers et al., 2003; Jensen et al., 2008), and nitrate does not co-exist with sulfide or elemental S (Luther et al., 1991; Konovalov et al., 2003; Çoban-Yildiz et al., 2006). However, the potential for S cycling in suboxic waters without the build up of sulfide has recently been demonstrated in the Chilean Oxygen Minimum Zone (Canfield et al., 2010), and DNA from potential sulfate reducers and sulfide oxidizers were found attached to large particulate matter in the Black Sea suboxic zone (Fuchsman et al., 2011).

In the southwestern Black Sea, water from the bottom layer outflow of the Bosporus Strait mixes with the overlying Cold Intermediate Layer forming the Bosporus Plume (Buessler et al., 1991; Murray et al., 1991; Ivanov and Samodurov, 2001). This plume enters the Black Sea as thin intrusions into the oxic, suboxic, and sulfidic layers (Oguz and Rozman, 1991; Konovalov et al., 2003). These intrusions inject oxygen, nitrate, and other oxidized species into the anoxic layers, where they are reduced. The rim current transports water affected by the Bosporus Plume along the coast to the east (Basturk et al., 1999; Konovalov et al., 2003; Poulain et al., 2005). From ratios of ammonium and sulfide, Konovalov and Murray (2001) calculate that 1.11 × 10^12^ mol of sulfide is missing from the Black Sea, and they attribute this loss to intrusions from the Bosporus Plume. They calculate that this approximates the re-oxidation of 50% of the sulfide production (Konovalov and Murray, 2001). Most of this sulfide oxidation is due to oxygen, but oxic intrusions also oxidize ammonium to nitrate/nitrite, which in turn can oxidize sulfide. Oxygen, nitrate, and nitrite intrusions were previously described at the mixing zone station in 2001 (Konovalov et al., 2003; Fuchsman et al., 2008). In this case, an intrusion of water from the Bosporus Plume created a second maximum in nitrate (up to 3.3 μM) at depths where sulfide is usually present (Fuchsman et al., 2008). The potential for autotrophic denitrification with sulfide is clearly present in the mixing zone of the Bosporus Plume in the Black Sea.

In this paper we provide evidence for autotrophic denitrification activity in the southwestern region of the Black Sea during an intrusion event of the Bosporus Plume, which caused overlap of ${\text{NO}}_{\text{x}}^{-}$ and sulfide. We examine likely denitrifying bacteria by comparing depth profiles of normalized TRFLP peak height from the mixing zone with the western central gyre.

## Materials and Methods

### Sampling

**Figure 1:**
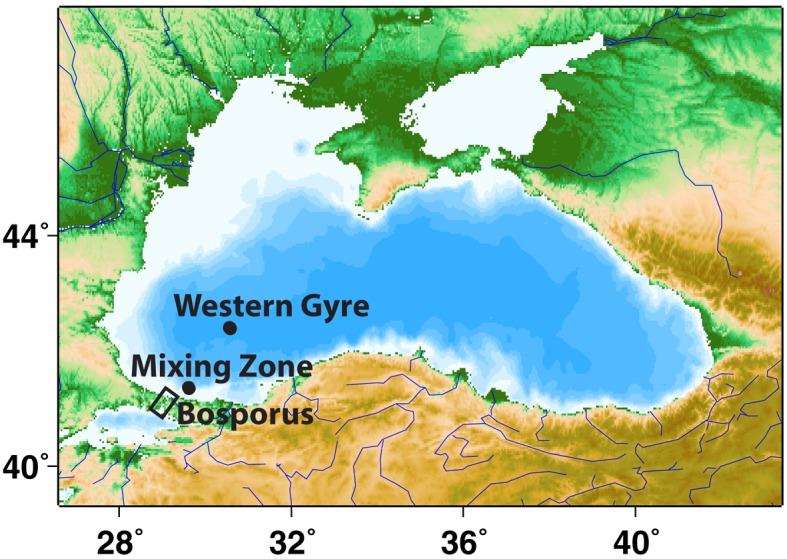
**A map of the Black Sea indicating the stations sampled in this study**. This map was made with Map-It (http://woodshole.er.usgs.gov/mapit).

Samples were collected using a CTD-Rosette with 10 L Niskin bottles and Sea-Bird sensors on three separate cruises in the Western Gyre of the Black Sea: (1) May 2001 at station 6 Voyage 162 leg 16 of the *R/V Knorr* (42°31′ N, 30°43.5′ E), (2) April 2003 at station 19 on Voyage 172 leg 7 of the *R/V Knorr* (42°30′ N, 31°00′ E), and (3) late March 2005 at station 2 on cruise 403 of the *R/V Endeavor* (42°30′ N, 30°45′ E). Samples were also collected in the mixing zone where the Bosporus Plume enters the Black Sea (Figure 1) on two cruises: Station 20 in April 2003 (41°26′ N, 29°34′ E) and Station 5 in March 2005 (41°26′ N, 29°34′ E).

### Nutrient concentrations

Oxygen was measured with the classic Winkler method, and sulfide by iodometric titration (Cline, 1969). In both cases, reagents were bubbled with argon to avoid contamination by atmospheric oxygen. Nitrate, nitrite, and ammonium were analyzed shipboard using a Technicon Autoanalyzer II system (see Fuchsman et al., 2008). Nitrate was not analyzed when there was consensus that sulfide would be in the sample.

### DNA

For DNA samples, 2 L were filtered onto 0.2 μm Millipore Sterivex filters. Samples were immediately frozen and stored at −80°C. The DNA extraction protocol was adapted from Vetriani et al. (2003) and includes 8–10 freeze thaw cycles between a dry ice/ethanol bath and a 55°C water bath followed by chemical lysis with lysozyme and proteinase K.

### TRFLP

TRFLP profiles were obtained using universal bacterial primers 27F-FAM and 1517R (Vetriani et al., 2003). PCR products were amplified for 30 cycles with annealing temperature of 48°C using Fermentas PCR MasterMix. Purified PCR products (QiaQuick columns; Qiagen) were separately digested overnight with four restriction enzymes (*Hae*III, *Hpy*1881, *Msp*I, *Mnl*I) and immediately ethanol precipitated (Fuchsman et al., 2011). TRFLP data from the Western Gyre in 2005 are previously published in Fuchsman et al. (2011).

Planctomycetes-specific TRFLP profiles were obtained using primers 58F-FAM and 926R (Wang et al., 2002). Planctomycetes PCR products were amplified for 30 cycles with annealing temperature of 60°C. Purified PCR products (QiaQuick columns; Qiagen) were separately digested overnight with restriction enzymes *Hae*III, *Hpy*1881, and *Msp*I and immediately ethanol precipitated (Fuchsman et al., 2012). Planctomycetes-specific TRFLP data for the Western Gyre in 2001, 2003, and 2005 are previously published in Fuchsman et al. (2012).

In both cases, analysis was performed on a MegaBACE 1000 apparatus (Molecular Dynamics) at the University of Washington Marine Molecular Biotechnology Laboratory. Electrophoretic profiles were visualized with Dax software (Van Mierlo Software Consultancy, Netherlands).

TRFLP profiles were normalized by total peak height. If the height of a peak was below 0.3% of the total peak height, the peak was removed from further statistical analyses. TRFLP peaks were binned using frame shifting (Hewson and Fuhrman, 2006) with four frames at 0.5 bp intervals, and for each enzyme, a resemblance matrix was obtained using the Whitaker index, which takes abundance (peak height) into account (Fuchsman et al., 2011). The maximum similarity of the four frames was used to calculate the hierarchical cluster analysis (using the group average) with the Primer 6 program. Error in the resemblance matrix and significance level of the cluster diagram was determined using a Monte Carlo simulation of 50 replicates. We used the average error for both normalized peak height [±46 relative fluorescence units (rfu) where total peak height is 18,000 rfu] and base pairs (±0.08 bp) as determined by 16 sets of duplicate TRFLP profiles. The lowest similarity between Monte Carlo simulated replicates was 77% (Fuchsman et al., 2011). The average error for the Planctomycetes was ±83 rfu, where total peak height was 15,000 rfu, and ±0.06 bp as determined by 14 sets of duplicate TRFLP profiles (Fuchsman et al., 2012).

Due to the replicability of the relative peak heights and the lack of cloning bias (Rainey et al., 1994), and because each PCR was run under the same conditions with similar extracts from the same amount of material, we were able to compare the relative abundance of the same restriction fragment (i.e., peak height) among multiple samples. However, due to PCR bias (Polz and Cavanaugh, 1998), comparison of heights among different restriction fragments was avoided. In other words, we only compare the relative abundance of a single taxon across samples and never compare the abundances of different taxa. More than one bacterial species can produce the same restriction fragment size; however, by ensuring that the shape of a fragment’s relative abundance profile with depth must be supported by multiple enzymes, and by using a small bin size, that risk was reduced. Arguments supporting the similar use of fragment peak height in data from the Southern California time series station can be found in Steele et al. (2011).

Both TRFLP and pyrosequencing of the V6 region of 16S rDNA were obtained from the Western Gyre in 2005 (Fuchsman et al., 2011). Both pyrosequencing and TRFLP avoid cloning biases (Rainey et al., 1994), but still contain PCR biases (Polz and Cavanaugh, 1998; Huse et al., 2008). Despite the use of different primers, conclusions from TRFLP data and V6 tag sequences compare well and we can identify many of the same OTUs using both techniques (Fuchsman et al., 2011). Not only are depth profiles of individual OTUs similar between techniques, but similarity indices are also similar when only V6 pyrosequences with >1% relative abundance were used (Fuchsman et al., 2011).

Predicted fragment lengths for the phylotypes discussed here are shown in Table 1. TRFLP OTUs can represent a variety of taxonomic levels depending on the variability in the restriction sites among related phylotypes. Most of the TRFLP OTUs presented here represent a unique sequence or small group of very similar sequences. However, with the restriction enzymes used here *Arcobacter* clone BS098 (GU145483) has the same restriction sites as a wide range of *Arcobacter* members including *Arcobacter nitrofigilis* (L14627) and Black Sea sediment enrichment cultures (AJ271653-4) though they are not particularly closely related. In this paper, TRFLP OTUs are named after the phylotype that was present in the V6 pyrosequence data from the Western Gyre in 2005 (Fuchsman et al., 2011). Full length clones representing these phylotypes were amplified with TRFLP primers and digested with the restriction enzymes (Fuchsman et al., 2011). The actual length of digested clones often differ slightly from the lengths predicted *in silico*. Identifying TRFLP peaks with a database of digested clones greatly improves the reliability of peak identification. Unfortunately, we do not have a digested full length clone representing *Arcobacter* and the *in silico* prediction deviates slightly from the observed peaks (Table 1).

**Table 1:** **Taxonomy and predicted fragment lengths for the phylotypes discussed in this study**.

ID	Taxonomy	Accesssion	*Hae*III	*Hpy*1881	*Msp*I	*Mnl*I	Primer
JK200	*Scalindua*	DQ368308	236	530	259	NA	58F-926R
BS142	WS3	GU145525	206	–––	297	144	27F-1512R
BS149	WS3	GU145532	206	–––	312	144	27F-1512R
BS129	BS-GSO2	GU145512	–––	–––	165.3	139.2	27F-1512R
BS098	*Arcobacter*	GU145483	228	–––	S:474	S:134	27F-1512R
					T:477	T:140?	
BS134	SAR324	GU145517	406	–––	160.9	134	27F-1512R
BS139	*Sulfurimonas*	GU145522	–––	–––	465	130	27F-1512R
BS077	SUP05	GU145462	193	–––	144.2	182.8	27F-1512R
BS007	SAR11 II	GU145392	292	–––	147	121	27F-1512R
BS110	Marine group A	GU145495	227	290	450	286	27F-1512R
BS040	*Cytophaga*-like	GU145425	410	–––	90	–––	27F-1512R

*16S rRNA clone PCR products were previously digested with all restriction enzymes and used to identify TRFLP peaks with a range ±0.5 bp from the length of the digested clone (Fuchsman et al., 2011) except for *Arcobacter*. Dashed line indicates that the enzyme did not cut the sequence within the size range examined (550 bp). NA stands for not applicable, S for *in silico*, and T for in TRFLP profile*.

### Autotrophic denitrification activity experiments

Samples for experiments were collected in 2005 at the mixing zone station at the shallowest depth where sulfide was detected (σ_θ_ = 16.4; 192 m), 5 m below that depth (σ_θ_ = 16.46; 197 m) and 20 m below (σ_θ_ = 16.52; 212 m) as well as from a depth where no sulfide was detected (σ_θ_ = 16.26; 178 m). Water was collected directly into 12.5 mL exetainers after overflowing with five times the volume of water. Vials were capped without the presence of bubbles. ^15^N-labeled ${\text{NO}}_{\text{3}}^{-}$ was added (for final concentration of 27 μM) to duplicate samples from each depth. Samples and controls were incubated at 7°C for 48 h. Experiments were stopped by addition of HgCl_2_, and 6 mL of water was replaced by helium and equilibrated overnight. Samples were measured directly by a Finnegan Delta XL isotope ratio mass spectrometer using the Conflo system in the Stable Isotope Lab, School of Oceanography, University of Washington. After gases were measured, the remaining water was analyzed for nitrite and ammonium concentrations using the Technicon Autoanalyzer II.

## Results

**Figure A1:**
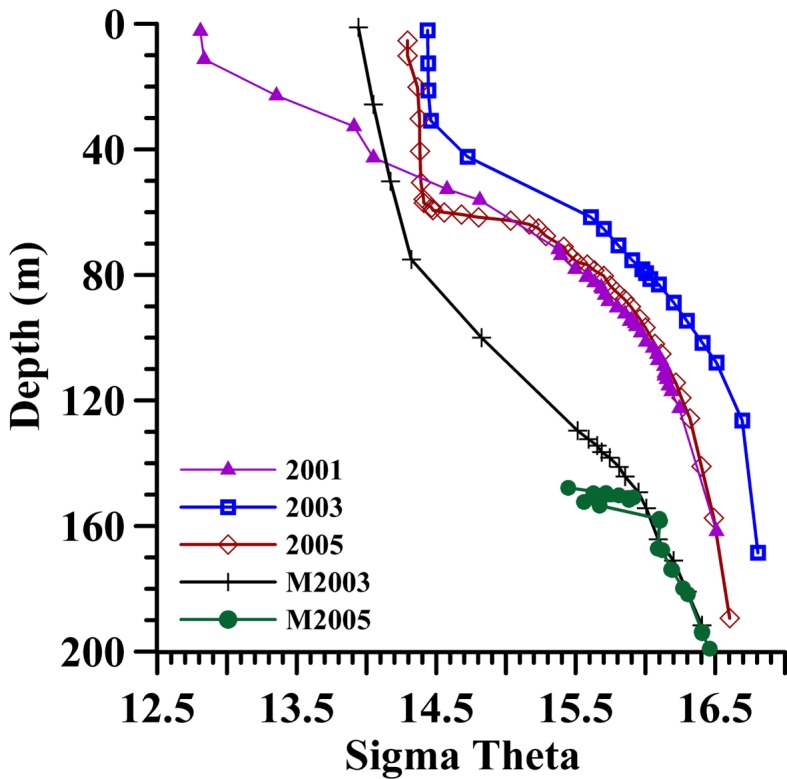
**The density (sigma theta) of the top 200 m for each station discussed in this paper: the Western Gyre in 2001 (purple triangles), 2003 (blue squares), and 2005 (red diamonds) and at the mixing zone station in 2003 (black crosses) and 2005 (green circles)**.

Due to the strong stratification of the Black Sea by salinity, characteristic inflections in the water-column profiles (such as nitrate) are generally associated with specific density values regardless of when and where they were sampled, but depths vary (Murray et al., 1995). Therefore, results presented here are plotted against potential density (σ_θ_) rather than depth (m). Densities occurred up to 75 m deeper at the mixing zone station than at the Western Gyre station and varied up to 15 m between years at the Western Gyre (Figure A1 in Appendix).

### Chemistry

**Figure 2:**
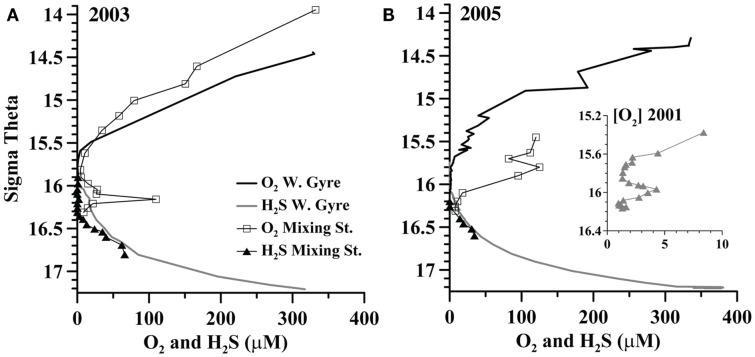
**A comparison between oxygen and sulfide in the Western Gyre and mixing zone stations in (A) 2003 and (B) 2005**. Inset: oxygen from the Western Gyre in 2001 during an intrusion event.

**Figure 3:**
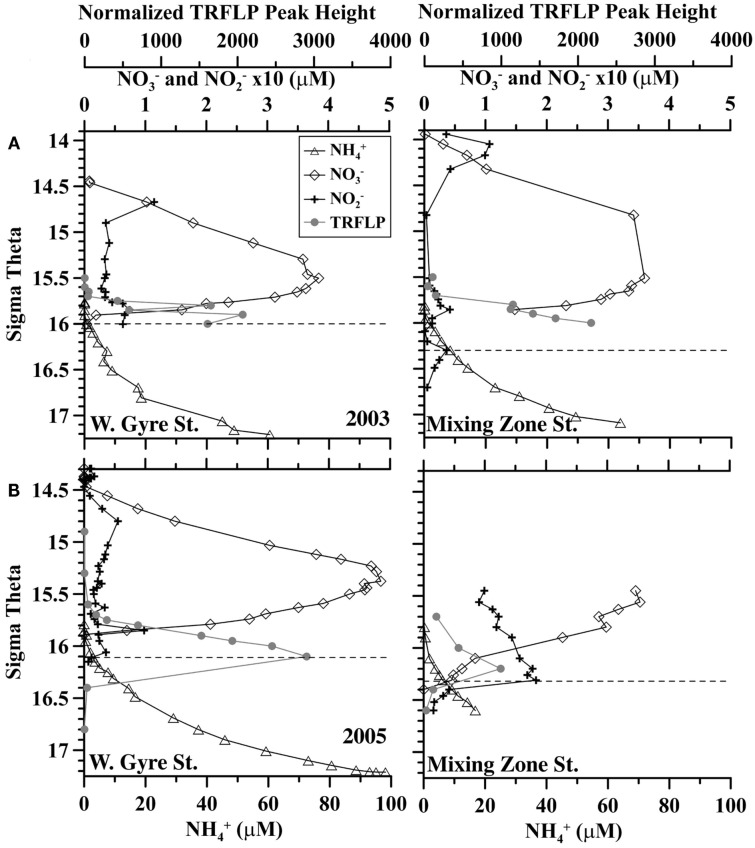
**Comparison of nitrate (diamonds), nitrite (crosses), ammonium (triangles), and *Scalindua* normalized TRFLP peak height (gray circles) between the Western Central Gyre and Mixing zone site in (A) 2003 and (B) 2005**. Shallowest detection of sulfide is indicated by dashed lines. Average error in normalized peak height is ±83 rfu.

A station where the Bosporus Plume enters and mixes with the Black Sea was occupied in late April 2003 and in March 2005 (Figure 1). Oxygen, sulfide, and nutrient data from this mixing station are compared to data from the Western Gyre in Figures 2 and 3. In 2003, the Western Gyre and Mixing Zone stations had similar oxygen profiles above σ_θ_ = 15.9, but there was an intrusion of oxygen at σ_θ_ = 16.15 at the mixing zone. In 2003, sulfide was first detected at σ_θ_ = 16.05 (81 m) in the Western Gyre, while in the mixing zone, sulfide became detectable above σ_θ_ = 16.4 (192 m; Figure 2). Nitrate maximum concentrations were similar between stations in 2003, and nitrite concentrations remained below 0.05 μM (Figure 3). In 2005, the oxygen concentrations in the mixing zone station were greatly elevated. Oxygen was measured down to σ_θ_ = 16.3 (7 μM, 180 m; Figure 2). Nitrate was 0.5 μM at σ_θ_ = 16.3 while nitrite had a maximum of 0.18 μM and was still elevated at σ_θ_ = 16.4 (192 m; Figure 3). Sulfide was not detected at 16.3 on the two casts at this station but was 12 μM just 6 m deeper on the cast not shown here. Given the detection limit for sulfide (3 μM), it is possible that nitrate and sulfide overlapped at this station. In any case, the flux of sulfide to σ_θ_ = 16.3 was 303 μmol m^−2^ day^−1^, so both nitrate and sulfide were available at this depth.

In the Western Gyre, oxygen extended deeper in the water column in 2005 than in other years. The top of the suboxic zone, defined as <10 μM O_2_, was at σ_θ_ = 15.38 (72 m) in 2001, σ_θ_ = 15.6 (61 m) in 2003, and σ_θ_ = 15.65 (78 m) in 2005. Higher oxygen concentrations at the Western Gyre in 2005 are due to a transitory lens of colder, more highly oxygenated water that appeared in the σ_θ_ = 15.4–15.6 range. On March 29th and 30th, the lens of more oxygenated water increased to 22 μM oxygen at σ_θ_ = 15.6 and then decreased to 12 μM at σ_θ_ = 15.6. When microbial samples were collected in 2001, oxygen concentrations (Figure 2) decreased from the top of the suboxic zone to near the detection limit at σ_θ_ = 15.85 and then increased to 2–4 μM from σ_θ_ = 15.92–16.05.

Ammonium was consistently present in the lower suboxic zone at both stations and during all years. However ammonium fluxes at σ_θ_ = 16.0 (calculated with diffusion coefficients from Ivanov and Samodurov, 2001) varied between stations and years with the lowest fluxes (190 μmol m^−2^ day^−1^) at the mixing zone station in 2003 and the western gyre in 2001 and the highest fluxes (330 and 310 μmol m^−2^ day^−1^) from the mixing zone station in 2005 and from the western gyre in 2003. In 2005, the Western Gyre in 2005 had an intermediate flux (270 μmol m^−2^ day^−1^).

### Evidence for autotrophic denitrification

**Figure 4:**
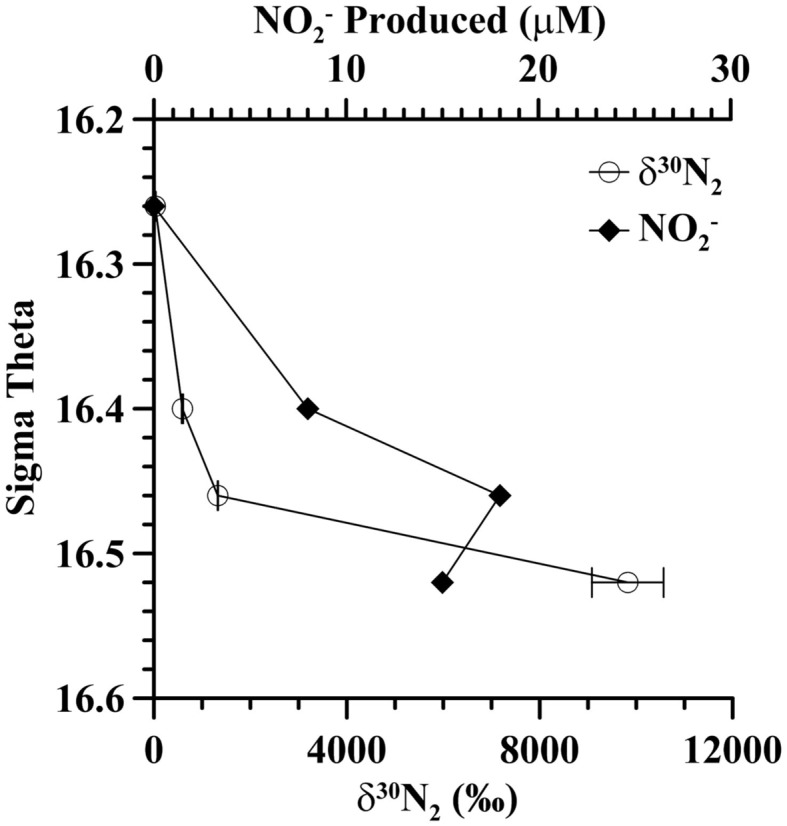
**Autotrophic denitrification activity at the mixing zone station in 2005 as indicated by ^30^N_2_ production (circles) during incubations with ^15^N-labeled nitrate**. Nitrite was also formed during the incubation (diamonds). Error bars indicate duplicate measurements.

In incubation experiments at the mixing zone station in 2005, where ^15^N-labeled ${\text{NO}}_{\text{3}}^{-}$ was added, enriched δ^30^N_2_, indicative of denitrification, was found in all sulfidic samples but δ^30^N_2_ was not enriched in the non-sulfidic sample [σ_θ_ = 16.26 (178 m; Figure 4)]. The amount of enriched N_2_ increased with depth from an enrichment of 590%_°_ at σ_θ_ = 16.4 (192 m) to an enrichment of 9800% at σ_θ_ = 16.52 (212 m). In the ^15^N-nitrate enriched samples from the sulfidic zone, there was substantial build up of nitrite, up to 18 μM at σ_θ_ = 16.46 (197 m; Figure 4) while ammonium concentrations were not different from controls.

Formation of δ^29^N_2_ from ^15^N − $\text{N}\text{-}{\text{NO}}_{\text{3}}^{-}$ enriched experiments is usually used to denote anammox activity. No enrichment in δ^29^N_2_ was detected in the non-sulfidic sample (σ_θ_ = 16.26), though ammonium was available from the ambient water, indicating that the anammox process was not occurring at that depth even though nitrite and ammonium were present. In the sulfidic zone, δ^29^N_2_ was enriched by 1%_°_ compared to controls. The relative abundance of *Candidatus Scalindua*, the genus of anammox bacteria in the Black Sea (Kirkpatrick et al., 2006), was low but present at σ_θ_ = 16.4 (Figure 3). However, the enrichment in δ^29^N_2_ could also be from denitrification using small amounts of unspiked ${\text{NO}}_{\text{X}}^{-}$ or N_2_O.

### Bacterial community

**Figure 5:**
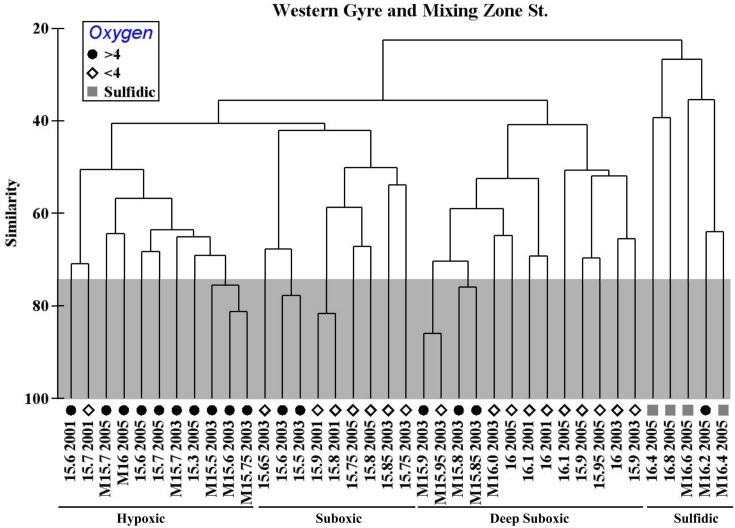
**Hierarchical cluster of the bacterial community as determined by TRFLP data (*Msp*I) from Western Gyre in 2001, 2003, and 2005 and Mixing Zone Site in 2003 and 2005**. Samples are named by the sigma theta and year where they were obtained. M indicates mixing zone station. Symbols indicate oxygen concentrations: >4 μM O_2_ (circle), <4 μM O_2_ (diamond), sulfidic (square). Error was determined using a Monte Carlo simulation; nodes within the gray box are not significantly different (e.g., σ_θ_ = 15.5–15.75 from the 2003 mixing zone site).

A Spearman Rank correlation between environmental ([O_2_], [H_2_S], [${\text{NO}}_{\text{3}}^{-}$], [${\text{NH}}_{\text{4}}^{-}$], [${\text{NO}}_{\text{2}}^{-}$], [PMn]) and biological (TRFLP) data indicated that a combination of nitrate, ammonium, and particulate manganese best explained all the bacterial data (*R* = 0.698). Oxygen was not found to be a significant factor, likely because samples with 82 and 5 μM oxygen have similar bacterial communities. However, if instead, the samples are binned into groups >4 μM oxygen, <4 μM oxygen, and sulfidic (see symbols in Figure 5), an ANOSIM analysis indicates that oxygen is a significant factor determining the differences in the bacterial communities (*R* = 0.460, *p* = 0.001). In a pairwise test, the >4 μM oxygen and <4 μM oxygen communities were different (*R* = 0.316, *p* = 0.001). The bacterial community does not seem to react linearly to oxygen, but instead to have a threshold.

**Figure 6:**
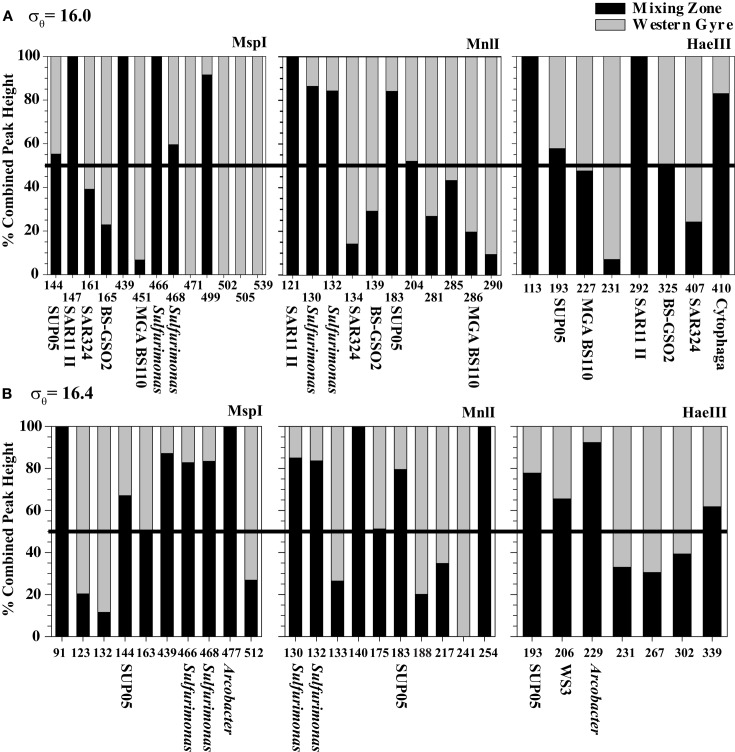
**A comparison of TRFLP chromatograms from (A) σ_θ_ = 16.0 and (B) σ_θ_ = 16.4 for three restriction enzymes at the Mixing Zone Station (black) and the Western Gyre (gray) where the *x*-axis displays different TRFLP peaks and the *y*-axis is the percent of peak height associated with each chromatogram**. The black line at 50% indicates where the relative peak height is the same at each station. Only peaks with normalized peak height above 3% of the total peak height are shown.

The community at the mixing site and the central gyre were fundamentally similar. At some depths, the community at the mixing zone has >60% similarity with the Whittaker index to communities at the western central gyre (Figure 5). However, there are some significant differences, especially in the sulfidic samples. We directly compared mixing zone station samples from 2005 to the same density at the central gyre station at two depths using the *Msp*I, *Mnl*I, and *Hae*III restriction enzymes with a cutoff of 3% total peak height to reduce noise (Figure 6). At σ_θ_ = 16.0, a density surface which contained no oxygen in the Western Gyre (97 m) and 47 μM oxygen at the mixing zone station in 2005 (166 m), the relative abundance of SAR11 clusters II phylotype BS007 was greater in the mixing zone station (Figure 6). The relative abundance of potential sulfur cycling bacteria *Sulfurimonas* phylotype BS139 and SUP05 BS077 phylotype along with unidentified *Msp*I peak 498 was also greater at the mixing zone station. The *Hae*III enzyme does not cut *Sulfurimonas* phylotypes, allowing Cytophaga phylotype BS040 to creep above the 3% total peak height threshold for that enzyme. At σ_θ_ = 16.4, a density surface which is in the sulfate reduction zone in the Western Gyre (141 m) but where autotrophic denitrification was detected at the mixing zone station (192 m), potential S oxidizers SUP05 BS077, *Sulfurimonas* BS139, *Arcobacter* BS098, and unidentified *Msp*I peak 91/Mnl peak 254 pair had much higher normalized peak height in the mixing zone station compared to the Western Gyre (Figure 6). For the *Hae*III enzyme, peak 206 representing the Black Sea WS3 group and unidentified peak 339 also had higher normalized peak height for the mixing station. The 206 cut site from *Hae*III represents multiple WS3 phylotypes that are separate peaks when different restriction enzymes were used. Two WS3 phylotypes have been identified by TRFLP in the sulfidic zone of the Black Sea (Fuchsman et al., 2011, supplemental).

**Figure 7:**
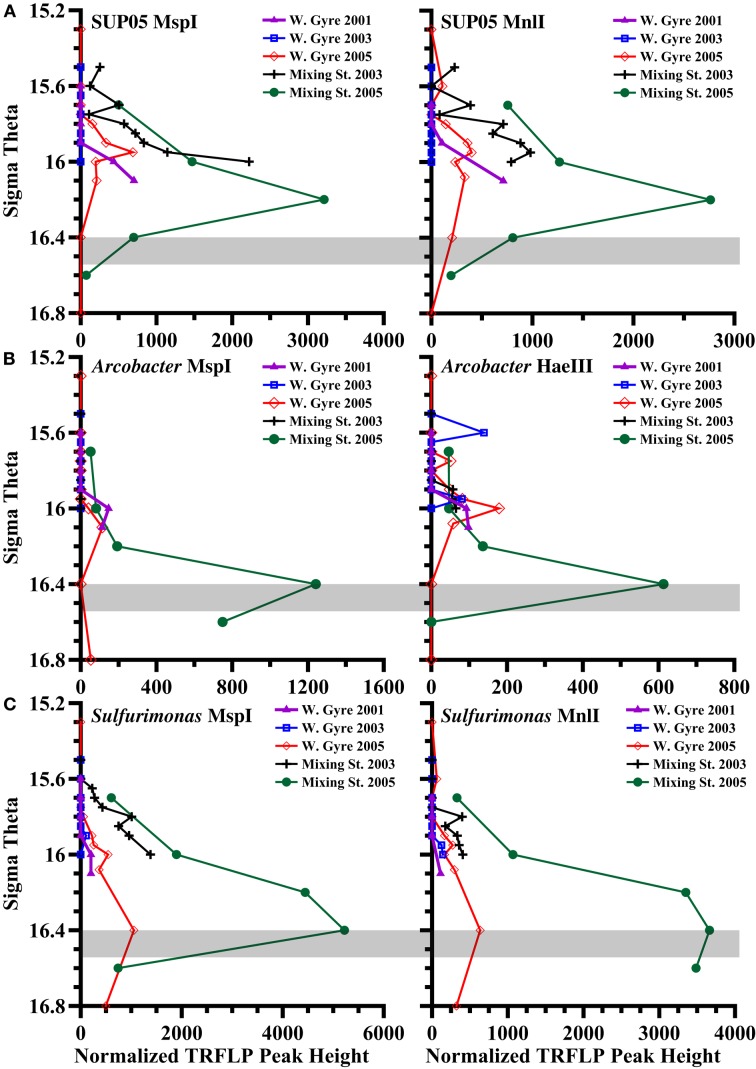
**TRFLP peak height depth profiles of autotrophic denitrification candidates that are enriched at the mixing zone station in 2005, obtained using multiple restriction enzymes, for the five stations: (A) Black Sea SUP05 phylotype BS077, (B) *Arcobacter* phylotype BS098, (C) Black Sea *Sulfurimonas* phylotype BS139**. The gray box indicates the range of autotrophic denitrification activity seen in the Mixing Zone station in 2005 (Figure 4).

**Figure 8:**
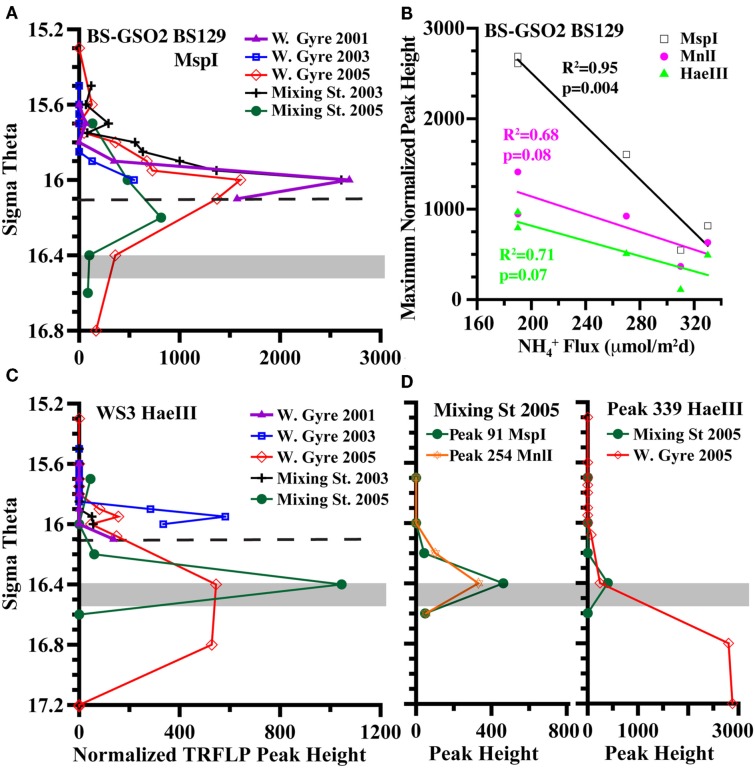
**Examination of autotrophic denitrification candidates (A) TRFLP peak height depth profiles of BS-GSO2 phylotype BS129, (B) the inverse correlation of BS129 peak height with ammonium fluxes at σ_θ_ = 16.0 at all five stations with a comparison between restriction enzymes, (C) TRFLP peak height depth profiles of WS3, and (D) depth profiles of unidentified peak 91 from digestion with *Msp*I and peak 254 from digestion with *Mnl*I and peak 339 from digestion with *Hae*III**. Dashed line indicates a typical depth for the shallowest detection of sulfide in the Western Gyre. The gray box indicates the range of autotrophic denitrification activity seen in the Mixing Zone station in 2005 (Figure 4).

When we look at all profiles over five stations instead of just the two depths in 2005, we see that the relative abundances of BS139 from the *Sulfurimonas* genus of epsilonproteobacteria, BS077 from the SUP05 group of gammaproteobacteria, BS098 from the *Arcobacter* genus of epsilonproteobacteria, and unidentified *Msp*I peak 91/Mnl peak 254 pair are clearly greater in the mixing zone station (Figures 7 and 8). Peaks 91/254 are in fact only seen in the mixing zone station in 2005 (Figure 8). Contrastingly, both group WS3 and peak 339 (*Hae*III) have maxima in the sulfidic zone in the Western gyre (Figure 8), implying that their presence at the mixing zone station is not due to the intrusions there.

This dataset also allows us to examine variability in the bacterial community within the Western Gyre. Upper suboxic zone samples (σ_θ_ = 15.5–15.7) from the Western Gyre in 2003 are included in the Suboxic cluster while upper suboxic zone samples from 2001 and 2005 are included in the Hypoxic cluster (Figure 5), perhaps due to the influence of oxygen intrusions in 2001 and 2005. In the deep suboxic zone, samples from 2001 clustered in a separate subcluster from samples from 2003 and 2005 (Figure 5). There was an oxygen intrusion into the deep suboxic zone in 2001 (Figure 2) and the particulate manganese maximum was also deeper. The maximum in particulate manganese at the Western Gyre varied σ_θ_ = 16.05 in 2001 (Konovalov et al., 2003) to σ_θ_ = 15.8–15.85 in 2003 and 2005 (Trowborst et al., 2006; Fuchsman et al., 2011).

### Planctomycetes community

The members of the Planctomycetes community at the mixing station were similar to those described in Fuchsman et al. (2012) with 60–70% community similarity to samples from the Western Gyre. *Msp*I peak 263, representing WS3 bacteria, had high peak height in the sulfidic mixing zone samples from 2005, corroborating information from the bacterial primers. At the mixing zone station, *Scalindua* peak height increased with depth starting at σ_θ_ = 15.7 in 2003, while in 2005 *Scalindua* peak height had a maximum at σ_θ_ = 16.2 (Figure 3). At σ_θ_ = 16.0, in the mixing zone station, *Scalindua* normalized peak height was significantly lower in 2005 than in 2003 (Figure 3).

The chemical parameters that most strongly correlated with the Planctomycetes community similarity among samples (Spearman Rank correlation) were nitrate, ammonium, and sulfide (*R* = 0.730 when combined), but ammonium and nitrate without sulfide explained most of the data (*R* = 0.729). Oxygen concentration was not found to be an important variable. However, the bacterial communities generally cluster by the presence of >3 or <3 μM oxygen or sulfide (ANOSIM *R* = 0.386, *p* = 0.001) supporting the importance of an oxygen threshold.

## Discussion

In the southwestern coastal Black Sea, intrusions from the Bosporus Plume inject oxygen, nitrate, and other oxidized species into the sulfidic layer (Konovalov et al., 2003). In 2005, there was abundant evidence of intrusions at the mixing zone station. Oxygen, usually only measurable to around σ_θ_ = 15.8, was measured down to σ_θ_ = 16.3 (Figure 2). Nitrate and nitrite were also unusually elevated at σ_θ_ = 16.3 and nitrite concentrations were still elevated at σ_θ_ = 16.4 (Figure 3). Sulfide was not detected at 16.3 (detection limit 3 μM), but the flux of sulfide to σ_θ_ = 16.3 was 303 μmol m^−2^ day^−1^, so both nitrate and sulfide were available. In experiments at the mixing zone station in 2005, where ^15^N-labeled ${\text{NO}}_{\text{3}}^{-}$ was added, enriched δ^30^N_2_ was found in all sulfidic samples but not in the non-sulfidic sample (σ_θ_ = 16.26; Figure 4). This implies autotrophic denitrification activity with sulfide as an electron donor. If we convert these enrichments to experimental rates, they range from 4 nM N day^−1^ at σ_θ_ = 16.4 to 10 nM N day^−1^ at σ_θ_ = 16.46 and 78 nM N day^−1^ at σ_θ_ = 16.52. These rates are an order of magnitude lower than experiments with comparable nitrate concentrations in Mariager Fjord (Jensen et al., 2009). These experimental rates do not represent *in situ* rates because nitrate additions (27 μM) were much higher than the largest values seen *in situ* (~3 μM; Fuchsman et al., 2008). Additionally, the positive dependence of autotrophic denitrification on sulfide concentration and the large accumulation of nitrite in the experiments (18 μM) are both consistent with trends seen in Mariager Fjord, Denmark (Jensen et al., 2009) and may be due to sulfide limitation. The accumulation of nitrite could also be due to the slower kinetics of nitrite reduction compared to nitrate reduction (Jensen et al., 2009) or to bacteria that merely perform the first step of nitrate reduction (Zumft, 1997). However, the consumption of five moles of sulfide for every two moles of nitrate (Jensen et al., 2009) indicates that sulfide limitation was likely in all of the experiments, but would have been especially important in the σ_θ_ = 16.4 experiment (14 μM H_2_S). While not indicating *in situ* rates, these experiments do indicate the ability of the bacterial community in the sulfidic zone of the mixing station to reduce nitrate when it becomes available, likely through intrusions from the Bosporus Plume.

Though the highest denitrification activity was seen at σ_θ_ = 16.52 with the addition of nitrate (Figure 4), it seems more likely that *in situ* rates at the time of sampling were higher between σ_θ_ = 16.3 and 16.4 where *in situ* nitrate/nitrite were naturally present (Figure 3). We also have DNA samples from σ_θ_ = 16.4. The relative abundances of BS139 from the *Sulfurimonas* genus of epsilonproteobacteria, BS077 from the SUP05 group of gammaproteobacteria, BS098 from the *Arcobacter* genus of epsilonproteobacteria, and unidentified *Msp*I peak 91/Mnl peak 254 pair are all clearly greater at σ_θ_ = 16.4 of the mixing zone station in 2005 compared to the Western Gyre (Figure 6). Members of the *Candidatus* genus *Scalindua*, known to mediate the anammox reaction but typically missed by universal bacterial primers, were also present at σ_θ_ = 16.4 at the mixing zone. Additionally, a labeled bicarbonate stable isotope probing experiment at the chemosynthesis maximum in the upper sulfidic zone of the central Black Sea in 2007, attributed autotrophic activity not only to members of the genus *Sulfurimonas* and the SUP05 (Glaubitz et al., 2010), found to be enriched in the mixing zone station in this study, but also to members of the BS-GSO2 group of gammaproteobacteria (Glaubitz et al., 2010). In the following section we examine these six bacteria to determine which was the most likely to mediate N_2_ production in the mixing zone site in 2005.

### Anammox

Sequences of potential anammox bacteria in the Black Sea are of the *Candidatus* *Scalindua* genus (Kirkpatrick et al., 2006). In the mixing zone station in 2005, *Scalindua* peak height had a maximum at σ_θ_ = 16.2 (Figure 3). This maximum was much reduced from the maximum at the same station in 2003 and from the Western Gyre in 2005 (Figure 3). Anammox activity was not detected at the mixing zone station in 2005, though only one depth was examined. However, that depth did correspond to the maximum in *Scalindua* peak height. *Scalindua* DNA at the mixing zone station may be remnants from previous activity, or *Scalindua* may be mediating Fe or Mn oxide reduction (van de Vossenberg et al., 2008).

### SUP05

A metagenome of SUP05 bacteria from Saanich Inlet, a seasonally anoxic fjord on Vancouver Island, Canada indicated that the SUP05 group of gammaproteobacteria had the ability to autotrophically oxidize sulfur compounds and also contain genes for the production of N_2_O (Walsh et al., 2009). Subsequently, SUP05 phylotypes were shown to be autotrophic in the upper sulfidic zone of the central Black Sea (Glaubitz et al., 2010). Transcripts of sulfur oxidizing genes from the SUP05 group have also been detected in the Chilean Oxygen Minimum Zone (Stewart et al., 2012). Altogether, this evidence could suggest a potential for autotrophic denitrification. Normalized TRFLP peak height for BS077 (the dominant SUP05 phylotype in the Black Sea) was much greater in the mixing zone stations than in the Western Central Gyre stations. However, phylotype BS077 had a maximum peak height at σ_θ_ = 16.2 in the 2005 mixing zone station, which was shallower than the depths where autotrophic denitrification was detected (Figure 7).

### BS-GSO2

A second group of uncultured gammaproteobacteria, BS-GSO2, was implicated in autotrophic activity in the upper sulfidic zone of both the Black and the Baltic Seas (Glaubitz et al., 2009, 2010) and linked to autotrophic denitrification in the Benguela upwelling zone (Lavik et al., 2009). Autotrophic activity in these sulfidic zones implies this group might be involved in sulfur oxidation (Glaubitz et al., 2009, 2010). BS129 (the dominant BS-GSO2 phylotype in the Black Sea) was identified in all years and stations but its relative abundance was greater in the Western Gyre than in the mixing zone station in 2005 (Figure 8). At the mixing station in 2005, BS129 had a maximum peak height at σ_θ_ = 16.2, and its relative peak height was greatly reduced at σ_θ_ = 16.4, where autotrophic denitrification activity was detected (Figure 8). In fact, the normalized peak height for BS129 at σ_θ_ = 16.0 appears to be anti-correlated with ammonium flux (Figure 8; *p* = 0.004 for *Msp*I). Normalized TRFLP peak height was greatest at the mixing zone station in 2003 and the Western Gyre in 2001 where ammonium fluxes were lowest. BS129 peak height was lowest in the Western Gyre in 2003 and the mixing zone station in 2005, both of which had high ammonium fluxes. Interestingly, an unknown gammaproteobacterium was found to mediate ammonium oxidation in the lower suboxic zone (Lam et al., 2007). Therefore, considering its correlation with ammonium fluxes, depth profile (Figure 8), and ability to fix carbon (Glaubitz et al., 2010), BS129 seems a likely candidate for autotrophic ammonium oxidation, but not autotrophic denitrification.

#### Arcobacter

**Figure 9:**
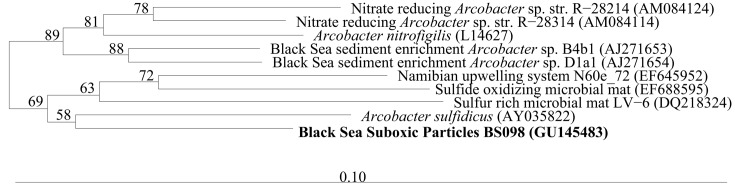
**A bootstrapped (1000) neighbor joining phylogenetic tree of relevant members of the *Arcobacter* genus was created in arb after aligning to a master database using NAST (greengenes.lbl.gov)**. The *Arcobacter* sequence from the Black Sea suboxic zone is in bold.

In the Benguela upwelling zone the epsilonproteobacteria *Arcobacter* were found at depths where autotrophic denitrification occurred (Lavik et al., 2009). *Arcobacter sulfidicus* has been found to autotrophically oxidize sulfide with oxygen forming elemental S mats (Wirsen et al., 2002; Sievert et al., 2007). *Arcobacter* phylotypes have been associated with such mats at sulfidic/oxic boundaries at hydrothermal vents, cold seeps, and the sediment water interface (Taylor and Wirsen, 1997; Moussard et al., 2006; Grunke et al., 2011). The *Arcobacter* depth profile in the Black Sea (Figure 7) would be consistent with either autotrophic denitrification or microaerophilic sulfide oxidation. The cultured representative, *A. sulfidicus*, which is closely related to BS098 found in the Black Sea (Figure 9), is microaerophilic and is incapable of oxidizing sulfide with nitrate (Wirsen et al., 2002). Some *Arcobacter* species can reduce nitrate heterotrophically (Heylen et al., 2006) but these isolates are not closely related to BS098 (Figure 9). Other *Arcobacter* enrichment cultures from sediments, such as Black Sea sediments (Thamdrup et al., 2000), have been found to reduce manganese oxides with acetate (Vandieken et al., 2012). The maximum in particulate manganese at the mixing zone station in 2005 is at σ_θ_ = 16.3 (B. Tebo, personal communication). The versatility of the *Arcobacter* genus makes predictions of the activity of the species at the mixing zone station particularly difficult.

#### Sulfurimonas

Members of the *Sulfurimonas* genus of Epsilonproteobacteria have been associated with autotrophic denitrification in the marine environment; many known strains of *Sulfurimonas* from hydrothermal vents and marine sediments can carry out autotrophic denitrification (Gevertz et al., 2000; Takai et al., 2006), and environmental clones affiliated with the genus have been extracted from marine sediments and correlated with active autotrophic denitrification (Shao et al., 2009; Zhang et al., 2009). *Sulfurimonas* phylotype GD17 has been found to mediate autotrophic denitrification in the Baltic Sea (Brettar et al., 2006). Black Sea sequences are closely related to GD17 from the Baltic Sea as well as to *Sulfurimonas denitrificans* (Glaubitz et al., 2010; Fuchsman et al., 2011). The normalized TRFLP peak height of *Sulfurimonas* phylotype BS139 was up to 10 times greater in the mixing zone stations than in the Western Central Gyre stations (Figure 7). The phylotype BS139 had a maximum peak height from σ_θ_ = 16.2–16.4 in the 2005 mixing zone station and still had significant abundance at σ_θ_ = 16.6 (Figure 7). Thus the *Sulfurimonas* peak spanned the depths where autotrophic denitrification activity was detected (Figure 4) and remains the leading candidate for mediating autotrophic denitrification.

**Figure 10:**
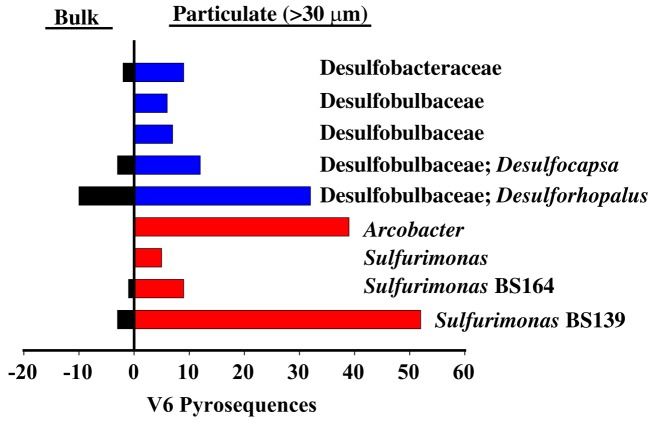
**Examples of potential sulfate reducers (blue) or sulfide oxidizers (red) enriched in particulate sample (30 μm filter) from σ_θ_ = 15.8 in the Western Gyre in 2005 compared to a bulk sample from the same depth, as determined from V6 pyrosequences (data from Fuchsman et al., 2011, supplemental)**.

*Sulfurimonas* was also enriched at σ_θ_ = 16.2. The presence of *Sulfurimonas* DNA at the Western Gyre site in 2005, 2007, and 1988 (Vetriani et al., 2003; Glaubitz et al., 2010) also implies *Sulfurimonas* can live at depths where sulfide is not detectable. There are two possible explanations for this. First, the depth profile for thiosulfate, another potential electron source for autotrophic bacteria (Takai et al., 2006) is unknown in the Black Sea during this time period. Second, pyrosequences of the V6 variable region of 16S rRNA identical to *Sulfurimonas* phylotype BS139 were also present in the particulate fraction in the suboxic zone in Western Gyre of the Black Sea, along with a *Desulfobacter* phylotype BS105 (GU145490) and pyrosequences from potential sulfate reducers from the Desulfobulbaceae and Desulfuromonadales families (Figure 10; data from Fuchsman et al., 2011). Many but not all cultured members of these families are sulfate reducers (e.g., Finster et al., 1994; Hoeft et al., 2004; Tarpgaard et al., 2006; Vandieken et al., 2006). *Arcobacter* V6 pyrosequences were also found on the particulate material and BS098 was sequenced from the particulates (Fuchsman et al., 2011). If *Sulfurimonas* phylotype BS139 or *Arcobacter* phylotype BS098 are indeed responsible for autotrophic denitrification in the mixing zone station, their presence on large particles in the nitrate-rich suboxic zone of the Western Gyre indicates that autotrophic denitrification may be fed by sulfate reduction inside sinking aggregates. This form of denitrification could easily have been missed in experiments by Jensen et al. (2008) due to the patchy nature of sinking particulate matter and the hydrodynamics of Niskin bottles (Altabet et al., 1992).

## Conclusion

Chemical profiles indicate that nitrate and sulfide may have co-existed at the mixing zone station in 2005 (Figures 2 and 3). ^15^N − ${\text{NO}}_{\text{3}}^{-}$ tracer experiments indicate autotrophic denitrification occurred in the sulfidic zone at this station (Figure 4). Though SUP05 and BS-GSO2 bacteria are autotrophic and have been found in sulfidic environments (Glaubitz et al., 2009, 2010; Lavik et al., 2009; Walsh et al., 2009), their depth profiles are not consistent with autotrophic denitrification at this station. Instead the depth profile for BS-GSO2 phylotype BS129 correlated with ammonium fluxes. In contrast, *Sulfurimonas* BS139, *Arcobacter* BS098, and unidentified *Msp*I peak 91/Mnl peak 254 pair have their greatest relative abundance in the zone where autotrophic denitrification was detected (Figure 7). Out of these three bacteria, we consider the Black Sea *Sulfurimonas* to be the most likely candidate for this denitrification because many *Sulfurimonas* species have previously been found to mediate autotrophic denitrification (Gevertz et al., 2000; Brettar et al., 2006; Takai et al., 2006). Evidence for the involvement of *Arcobacter* and peak 91 is less clear.

For most of the Black Sea, both anammox (Kuypers et al., 2003; Jensen et al., 2008) and heterotrophic denitrification (Fuchsman et al., 2008) are the important nitrogen loss pathways. Biogeochemical modeling indicates that autotrophic denitrification from overlapping depth profiles of nitrate and sulfide may only contribute 1% to nitrogen loss in the central Black Sea (Konovalov et al., 2008). However, where the Bosporus enters the Black Sea, autotrophic denitrification appears to be more important, and autotrophic denitrification associated with sinking particles has not yet been quantified. Future work should investigate these possibilities in order to better constrain the role of autotrophic denitrification in the Black Sea’s nitrogen cycle.

## Conflict of Interest Statement

The authors declare that the research was conducted in the absence of any commercial or financial relationships that could be construed as a potential conflict of interest.
